# Supplementary material for: From sequence to enzyme mechanism using multi-label machine learning
Source: BMC Bioinformatics. 2014 May 19;15:150. doi: 10.1186/1471-2105-15-150 (PMC4229970; doi:10.1186/1471-2105-15-150)
Supplement: Additional file 2 — Java code of ml2db. Additional file ml2db_code.tar.gz contains the Java source code to run the multi-label machine learning experiments and save the results to database. The code’s Javadoc is included. [file 1471-2105-15-150-S2.zip › additional file 2/ml2db/utils/doc/index-files/index-5.html]

E-Index


JavaScript is disabled on your browser.


- Overview
- Package
- Class
- Use
- Tree
- Deprecated
- Index
- Help

- Prev Letter
- Next Letter

- Frames
- No Frames

- All Classes

A B C D E F G H I J K L M N O P Q R S T U V W X Y 


## E

edu.cornell.lassp.houle.RngPack - package edu.cornell.lassp.houle.RngPack


elementExistsInTable(String, String, String, DbConn) - Static method in class uk.ac.standrews.utils.main.database.DbUtils
:   Get if an element exists in a table column

elementsExistsInTable(String, String, String, String, String, DbConn) - Static method in class uk.ac.standrews.utils.main.database.DbUtils
:   Get if two elements exists in the same table row

elementsToString() - Method in class uk.ac.standrews.utils.main.collectionutils.setutils.Set


elementsToString() - Method in class uk.ac.standrews.utils.main.setutils.Set


emptyKeys() - Static method in class uk.ac.standrews.utils.test.Data


emptyValue() - Static method in class uk.ac.standrews.utils.test.Data


END\_TIMESTAMP - Static variable in class uk.ac.standrews.utils.main.database.DbUtils


EntrezUtils - Class in uk.ac.standrews.utils.main
:   Utilities for Entrez services.

EntrezUtils() - Constructor for class uk.ac.standrews.utils.main.EntrezUtils


equals(TableColumn) - Method in class uk.ac.standrews.utils.main.database.TableColumn
:   Compares two columns by their name, returns true if the two columns have
    the same name, false otherwise.

equals(Object) - Method in class uk.ac.standrews.utils.main.diff.Difference
:   Compares this object to the other for equality.

error(Object) - Static method in class uk.ac.standrews.utils.main.LogUtils


error(Object, String) - Static method in class uk.ac.standrews.utils.main.LogUtils


error(String) - Static method in class uk.ac.standrews.utils.main.LogUtils


errorPopUp(String) - Static method in class uk.ac.standrews.utils.main.guiutils.GuiUtils
:   Error pop-up window with message (icon: stop)

eSummaryToString(XmlNode) - Static method in class uk.ac.standrews.utils.main.EntrezUtils
:   Test method to print an an Entrez eSummary xml report

evaluateCondition(XmlNode, String) - Static method in class uk.ac.standrews.utils.main.webutils.simpledomparser.XmlSearcher
:   Check whether the desired string is either a tag or text for this
    element.

evaluateCondition(XmlNode, String, String) - Static method in class uk.ac.standrews.utils.main.webutils.simpledomparser.XmlSearcher
:   Check whether the desired string is either a tag, text or an attribute
    \*value\* for this element.

executeQuery(String) - Method in class uk.ac.standrews.utils.main.database.DbReader
:   Executes an sql query returning a result set.

executeUpdate(String) - Method in class uk.ac.standrews.utils.main.database.DbWriter
:   Executes any SQL update which does not return results (create/drop table
    etc)

existsInTable(String, DbConn) - Static method in class uk.ac.standrews.utils.main.database.DbUtils


externalKeys() - Static method in class uk.ac.standrews.utils.test.Data


extractDoubleBetween0And1() - Static method in class uk.ac.standrews.utils.main.stats.UniformRandomUtils
:   Returns a random double between 0 and 1

extractInteger(int) - Static method in class uk.ac.standrews.utils.main.stats.UniformRandomUtils
:   Returns a random integer between 0 and size-1

extractMapEntry(Map, int) - Static method in class uk.ac.standrews.utils.main.maputils.MapUtils
:   Extracts a random entry (key-value association) from a map

extractUniformRandomMapEntry(Map) - Static method in class uk.ac.standrews.utils.main.stats.UniformRandomUtils
:   Extracts a random entry (key-value association) from a map

A B C D E F G H I J K L M N O P Q R S T U V W X Y

- Overview
- Package
- Class
- Use
- Tree
- Deprecated
- Index
- Help

- Prev Letter
- Next Letter

- Frames
- No Frames

- All Classes
